# The aversive bystander effect whereby egalitarian bystanders overestimate the confrontation of prejudice

**DOI:** 10.1038/s41598-023-37601-3

**Published:** 2023-06-29

**Authors:** Hanna Szekeres, Eran Halperin, Anna Kende, Tamar Saguy

**Affiliations:** 1 Department of Psychology, ELTE Eötvös Loránd University, Izabella Street 46, 1064 Budapest, Hungary; 2 Hebrew University, Jerusalem, Israel; 3 Reichman University (IDC), Herzliya, Israel

**Keywords:** Human behaviour, Social behaviour

## Abstract

Everyday expression of prejudice continues to pose a social challenge across societies. We tend to assume that to the extent people are egalitarian, they are more likely to confront prejudice—but this might not necessarily be the case. We tested this assumption in two countries (US and Hungary) among majority members of society, using a behavioral paradigm for measuring confronting. Prejudice was directed at various outgroup minority individuals (African Americans, Muslims and Latinos in the US, and Roma in Hungary). Across four experiments (N = 1116), we predicted and found that egalitarian (anti-prejudiced) values were only associated with hypothetical confronting intentions, but not with actual confronting, and stronger egalitarians were more likely to overestimate their confronting than weaker egalitarians—to the point that while intentions differed, the actual confronting rate of stronger and weaker egalitarians were similar. We also predicted and found that such overestimation was associated with internal (and not external) motivation to respond without prejudice. We also identified behavioral uncertainty (being uncertain how to intervene) as a potential explanation for egalitarians’ overestimation. The implications of these findings for egalitarians’ self-reflection, intergroup interventions, and research are discussed.

## Introduction

While in today’s democratic societies there is a decrease in violence and mass atrocities against minority groups, structural and everyday forms of racism still frequently occur and can quickly intensify. From time to time, we witness prejudice and discrimination towards minority individuals on the bus, in the grocery store, at school, in our workplace, or at gathering with friends or family. Occasionally, someone decides to intervene and stand up in face of prejudice. There are lay theories and assumptions regarding the question of who would confront prejudice^1^. We tend to assume that bystanders’ values impact their intervention tendency, such as their egalitarians beliefs that prejudice is wrong and should be controlled. However, this assumption may be subjected to correspondence bias, that is, when people encounter confronting events they infer a corresponding internal attribute, such that the confronter must be egalitarian. We propose that individuals’ own anti-prejudiced values set up expectations for confronting—but end up weakly predicting confrontation in the heat of the moment. This is problematic because individuals might delude themselves that their values would drive them to stand up against prejudice, and consequently would not self-reflect and prepare for situational factors that might stop them from doing so.

Prior studies reported that most individuals fail to confront when witnessing prejudice^2–4^, even though confronting is effective in decreasing prejudice in the perpetrator, especially if done by those who do not belong to the target group of prejudice^5,6^. Therefore, it is important to understand whether values impact bystander intervention, and if not, what may be an obstacle for taking such action. We tested this inquiry across various intergroup contexts, by examining hypothetical and actual confrontation of prejudice among majority members of society—where prejudice was targeted towards a minority group.

### Egalitarian values and confrontation of prejudice

Egalitarian values, like beliefs that inequality and prejudice is wrong and should be controlled, are shown to predict confronting intentions. Specifically, lower social dominance orientation (SDO; tendency to support hierarchy in society and inequality) was associated with willingness to act against prejudice directed at minorities among German high-school students^7^. Similarly, low SDO and political left-wing values were associated with German adults’ intention to confront prejudice on behalf of various stigmatized groups, like Arabs, People of Color, or gays^8^. Among majority Australian respondents politically left-wing values^9^ and higher internal motivation to respond without prejudice (IMS)^10^ were associated with bystander anti-racism intentions in support of Indigenous Australians.

However, these studies relied on self-reports and estimations, that is, they measured intentions to confront (i.e., hypothetical confronting), rather than actual behavior. Intentions to confront may not be an accurate reflection of real-life behavior as even targets of bias tend to overestimate their likelihood of confronting^11,12^. Individuals who are not the target of prejudice tend to feel discomfort and distress when witnessing prejudice^13^, especially those with egalitarian anti-prejudiced values (those high on IMS)^14^. At the same time, prior work found that while White Americans anticipated feeling upset at someone who expressed racism, when placed in the situation, they did not take the opportunity to socially reject the racist individual^3,15^. Similarly in a study measuring confronting specifically, heterosexual participants who imagined witnessing a homophobic slur reported significantly higher intentions of confronting the slur than did people who actually witnessed the slur^2^. Similarly, in the current research, we aimed to understand this discrepancy between having negative dispositions about such situations yet not confronting—by testing the role of egalitarian (anti-prejudiced) values in this process and predicting that egalitarian individuals are especially likely to overestimate their likelihood of confronting.

### Aversive racism: discrepancy between egalitarian values and action

While there is scarce research investigating the relationship between egalitarian values and actual confrontation as an outcome, there is evidence that intergroup attitudes are a weak predictor of prosocial intergroup action^16,17^. Specifically, the aversive racism framework suggests that holding egalitarian values does not guarantee helping the outgroup^18^. In a classic experiment, a Black or White confederate called registered White liberal and conservative voters for help (the callers’ ethnicity was identifiable through their voice). Interestingly, when the helping request was clear (and with that, the expected norm of helping), liberals helped the Whites and Black callers to the same amount—while conservatives discriminated the Black caller^19^. However, when the request for help was more ambiguous, liberals showed racial discrimination (by hanging up to a Black caller more readily than to a White caller), while conservatives showed no such bias (those who hung up early were equally likely to do so with White or Black callers). Similarly, in another study also testing actual behavior, highly prejudiced White student participants were as likely to help a Black victim (when something allegedly fell on them in another room) as low prejudiced students^20^.

These findings support aversive racism theory. Aversive racism is the biased behavior of well-intentioned majority members of society who endorse egalitarian values and consider themselves non-prejudiced^18,21–24^. Aversive racists would not act inappropriately when discrimination would be obvious to others, or themselves^24^. However, discrimination would occur in situations in which normative structure is weak, when the guidelines for appropriate behavior are vague, when the basis for social judgment is ambiguous, or when one can justify or rationalize a negative (or lack of) response by other factors than race, therefore maintain a non-prejudiced self-image and avoid guilt^18,25^. Aversive racism framework thus suggests that subtle, indirect, and ambiguous contexts may lead to more discrimination towards disadvantaged group members for advantaged group members who believe that they are not racist^21^.

### The internal and external motivation to avoid expressing prejudice

Beyond aversive racism, the justification-suppression model similarly contends that individuals in general are motivated to avoid showing racial discrimination (due to perceived suppressing social norms)—unless justification for acting prejudiced is available^26^. Individuals control prejudice either to avoid negative consequences of interpersonal (e.g., exclusion) or intrapersonal (e.g., guilt) nature^26,27^. In other words, individuals can be either externally or internally motivated to respond without prejudice^16,28,29^.

Some advantaged group members are motivated to avoid prejudice due to an internal desire to maintain an egalitarian identity and feel as they are binding to their egalitarian standards^29^. The aversive racism framework is focused mostly on these individuals with internal motivation to control prejudice^16,21,30^ contending that their unconscious biases may explain their occasional racist behavior^21,22,31^.

At the same time, aversive racists may be those who are driven by external motivations, who still hold explicit and conscious prejudice, but they have become more aware of social norms against it and are more guarded about public expressions of bias to convince others they are unprejudiced^29,32,33^. Indeed, externally motivated people were found to attempt to conceal and avoid appearing prejudiced^34,35^. Therefore, they would engage in subtle rather than blatant discrimination^18^. In the current research, we aim to test the role of internal and external motivation in the overestimation of confronting prejudice.

### The aversive bystander effect

In the present research, we apply the aversive racism and justification-suppression frameworks to situations of witnessing prejudice against a stigmatized minority group. Specifically, we test the extent to which egalitarian values predict confronting prejudice, by comparing actual vs hypothetical confronting. We propose an aversive bystander effect whereby egalitarian (anti-prejudiced) values predict hypothetical confronting of prejudice, but not actual confronting.

The context of witnessing prejudice breeds potential for manifestation of a phenomenon like aversive racism for one, because the situation is often not clear-cut (i.e., the bystander is not certain that the target needs help), especially if the target is not present, and indeed explicit request for help is not common in these scenarios. Secondly, because not confronting in this situation is an error of omission and not commission. While acting in an inappropriate way (commission) is a red flag for egalitarians, not acting when they should have (omission) is a more ambiguous situation that allows for rationalization and so inaction without harming a non-prejudiced self-concept.

To this end, most bystanders would overestimate the likelihood of confronting prejudice, but this overestimation would be especially emphasized among egalitarians, because they are motivated to believe they should and would confront and meanwhile they underestimate the power of a bystander situation. We further test the motivation involved in the aversive bystander effect and predict that egalitarians’ pronounced overestimation is not associated with external motivation, but with internal motivation to avoid prejudice.

### Bystander intervention: the role of uncertainty

The Confronting Prejudiced Responses model (referred to as CPR)^36^ builds upon the classic model of bystander intervention in physical emergencies^37^ to provide a comprehensive framework for understanding the challenges associated with confronting prejudice. According to the CPR, bystanders must recognize prejudiced behavior, perceive it as an urgent matter requiring immediate action, acknowledge their personal responsibility, and identify an appropriate response. Previous research employing a behavioral paradigm has demonstrated that confusion and difficulty in processing a situation diminish moral courage (e.g., intervening in a theft)^38^. Considering these findings, in our research, we also focused on the role of uncertainty, specifically behavioral uncertainty, which refers to the uncertainty experienced when attempting to identify an appropriate response upon witnessing prejudice^36^. Since situational ambiguities contribute to the strengthening of the aversive racism effect^18^, consequently they would reinforce the aversive bystander effect as well.

We proposed that uncertainty plays a role in explaining the tendency to overestimate one's willingness to confront prejudice, particularly among egalitarians. There are two reasons for this. Firstly, egalitarians may fall victim to the optimism bias, believing that their strong commitment to non-prejudice automatically renders them more prepared to respond to biased incidents. Meanwhile driven by their non-prejudiced motivation, they underestimate the power of the situation. Secondly, because when an event is appraised by individuals as threatening to their sense of self and they are uncertain about how to handle it, they may opt to disengage and distract themselves rather than take control^39,40^. For egalitarian bystanders, witnessing prejudice poses a threat to their unprejudiced self-concept, especially when they are unsure about how to respond. Consequently, this uncertainty would lead them to avoid involvement and confrontation.

### Overview of studies

To test the aversive bystander effect, we conducted four studies in two countries among the majority members of society with four different outgroups—African Americans in US in study 1a, Muslims in US in study 1b, Roma in Hungary in study 2, and Latinos in US in study 3. This was done to increase external validity of the findings.

The studies were conducted in two rounds (studies 1b-3). In the first round, we measured individuals’ anti-prejudiced egalitarian values, i.e., *outgroup-specific* Internal and External Motivation to Respond Without Prejudice (IMS and EMS)^29^. IMS reflects sincere and privately held ideals of oneself as a fair-minded person while EMS stems from perceived public social pressure to adopt nonbiased attitudes and behavior.^29^

In the second round, we randomly assigned participants to the actual vs. hypothetical conditions. To measure actual confronting, we developed a behavioral paradigm, in which participants were engaged in an online game. Participants witnessed another player’s prejudicial treatment of an outgroup minority, and then had an opportunity to confront the prejudiced player (actual condition). In the hypothetical conditions, participants also took part in this online game, but either did not witness any bias (Study 1a) or witnessed a different non-intergroup type of bias and had an opportunity to confront (Study 1b, Study 3), or witnessed the exact same prejudice but had no opportunity to confront (Study 2–3). To measure hypothetical confronting, following these control scenarios we described participants the prejudiced event that occurred in the actual confronting condition and asked them if they would have confronted in that situation.

Across all studies, we predicted that egalitarian anti-prejudiced values (IMS) would be more strongly associated with hypothetical than with actual confronting of prejudice. We hypothesized that both strong-egalitarians and weak-egalitarians would overestimate the likelihood of confronting (higher hypothetical than actual confronting). However, this effect would be especially emphasized among stronger egalitarians, who expect themselves to confront (when it is hypothetical) more than weaker egalitarians, but their confronting in the actual situation would not differ significantly from weak-egalitarians. We further predicted that overestimation would be associated with IMS, and not with EMS. In Study 3, we tested whether behavioral uncertainty (being uncertain how to respond) explains egalitarians’ overestimation. Data, analysis code, and supplemental materials are available at https://osf.io/d6m47/.

## Study 1a

### Method

#### Participants and procedure

We recruited 120 U.S. participants through mTurk. Respondents who identified as Black/African American/Afro-Caribbean or Latino/Hispanic at the beginning of the survey were directed to the hypothetical condition and their data were dropped from analyses (n = 22). We also excluded those who failed two attention check questions (e.g., “Please mark the answer that says somewhat disagree.”, n = 10) and those who figured out the purpose of the study (following questions of the game we asked: “Please describe what was the study about.”, n = 7). Following these exclusions, 82 participants remained in the study (60% female, age range: 18–61, *M*_age_ = 32.2 years, *SD*_age_ = 10.3), who were randomly assigned to an actual (n = 38) or a hypothetical confronting condition (n = 44). Sensitivity power analysis (using G*Power 3.1) indicated that z-test for two independent Pearson r’s with 80% power, α = 0.05, two-tails, are sensitive to effects from *z* = 1.96. Participation was anonymous and voluntary and written informed consent was obtained from all participants. The research was conducted with the IRB approval of Eötvös Loránd University (204/2016) in accordance with the Declaration of Helsinki.

In both conditions, participants believed that they were observing an online game, where players answer logic questions—which was pre-programmed. In the actual condition, participants witnessed that a White player unfairly eliminated a Black player from the game and messaged the participant with a prejudiced remark. To measure actual confronting, participants had an opportunity to reply to this biased player. In the hypothetical condition, participants witnessed no bias, but at the end of the study, we disclosed them the prejudiced event the way it occurred in the actual condition (allegedly as part of another participant’s report) and asked them the likelihood of them confronting in this situation. In this study, IMS-EMS was measured following the manipulation to conceal the purpose of the game.

Following demographic questions (on gender, age, race/ethnicity, relative socioeconomic status), the game stimuli appeared, which was then followed by filler questions about the game (e.g., outlook, flow), then a seemingly unrelated “sociology” survey about social issues appeared. In this latter section we included the IMS-EMS. In the hypothetical condition, the confronting intention measure was included at the end of the survey. Finally, all participants received written debriefing (see appendix on OSF).

#### IMS–EMS scale

We adapted the Internal and External Motivation to Respond Without Prejudice (concerning “Black people”) from Plant and Devine (1998). We asked participants their agreement (on 9-point scale) to 4 items for IMS (α = 0.95), and 4 items for EMS (α = 0.89). For full scale see appendix on OSF.

#### Witnessing paradigm (logic-IQ game)

**Figure 1:**
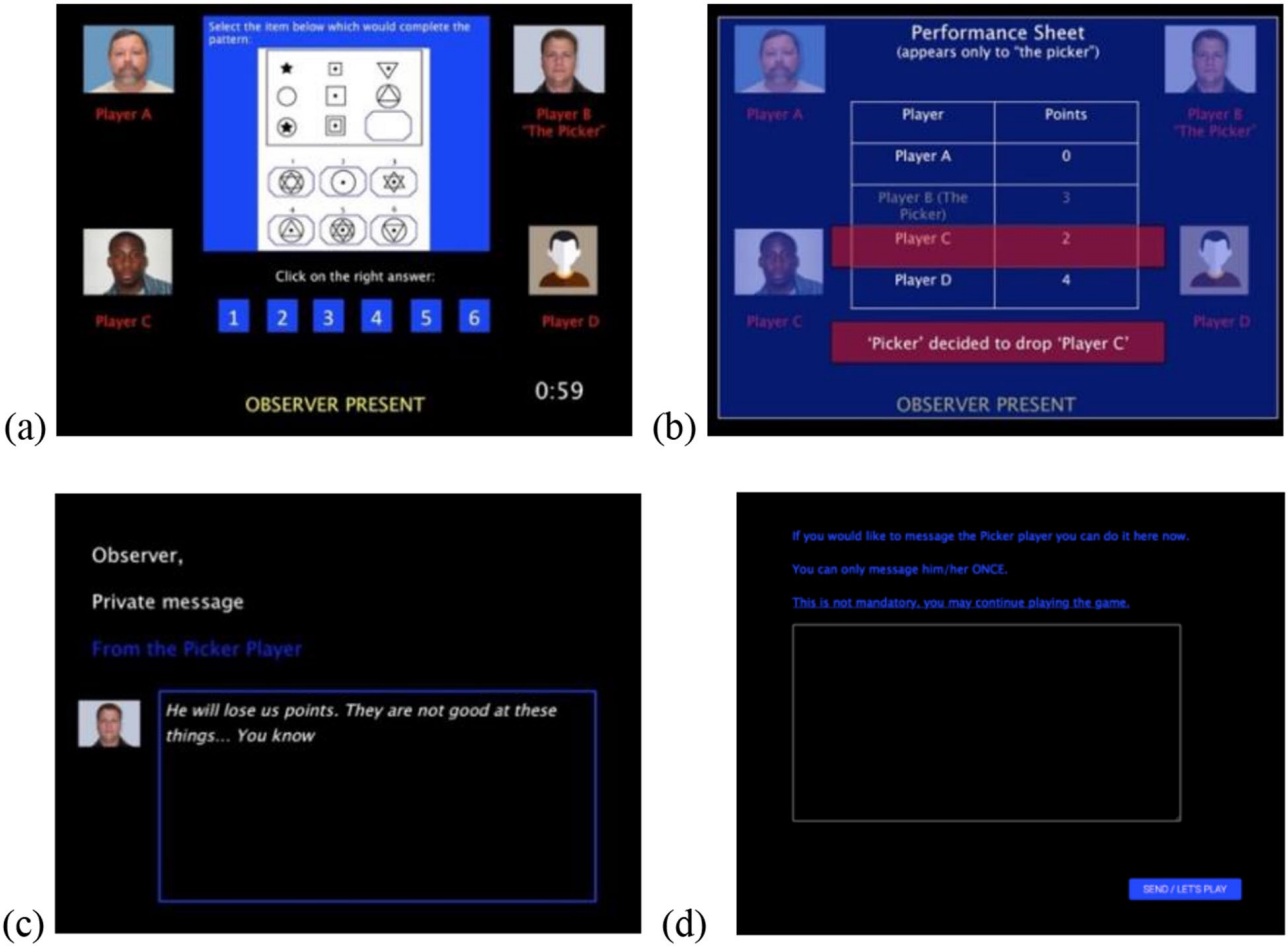
Scenes from the Logic-IQ game paradigm in Study 1a: (**a**) during a question posed to players; (**b**) performance sheet with players’ earned points and showing that Black player is eliminated by the prejudiced (Picker) player; (**c**) Picker player’s prejudiced message; (**d**) Message box providing an opportunity to respond to the prejudiced (Picker) player. Pictures taken from the Chicago Face Database (Ma, Correll, & Wittenbrink, 2015).

All participants believed they were observing real players in an online game (and then they would play it themselves). The game site was made to look like a different online platform. To conceal the purpose of the game, participants were told we test “how the presence of others facilitates or impairs performance”, and that their role of observers serve to increase the players’ feeling of being “exposed” during performance. Participants were first provided with description and instruction of the study and game (see appendix on OSF), where we included elements aimed to increase the credibility of our cover story. The game called “Logic IQ game” was described as composing of four players (each appearing with a pictogram or photo). The goal was to win as much money as possible by answering logic questions correctly as a group. In each group of players there is a designated established player (called the “Picker”), who eliminates one player at the end of each round (who then loses all his earned money). After elimination, all points (money) are divided equally between the three remaining players (thus, it is beneficial to keep better players and drop the weakest), and then the observer would join the game. Following instructions and “training”, participants were directed to a seemingly different online surface, where in reality they were exposed to a pre-recorded video (see Fig. 1).

All participants observed the game of three White players and one Black player (see Fig. 1a). During the game, participants received a decoy message from the Picker (aimed to decrease suspicion and to draw attention to the screen). After the last question, a performance sheet appeared with players and their earned points. To manipulate prejudice and discrimination, in the actual condition the Picker player eliminated the Black player (see Fig. 1b), who was *not* the weakest player, and then privately messaged the participant saying “He will lose us points. They are not good at these things… You know” (see Fig. 1c). In the hypothetical condition, the Picker player dropped the weakest (White) player and privately messaged the observer saying “He will lose us points. He is not performing well at all… You saw”. Following this message, for all participants, a text box appeared where they could message the Picker player (i.e., confront in the actual condition). After some delay, a system error message appeared, and the game ended before the participants could start to play themselves.

Note, we told participants at the beginning of the game that when they themselves join the game to play, they will play with the same Picker player they see in the observing phase. This was done to create some ramifications, costs for confronting.

#### Actual confronting

Confronting in the actual condition was measured by coding participants’ responses that they provided in the reply message box to the biased player. Three independent judges rated whether a response was ‘non-confronting’ or ‘confronting’, or ‘other’ (answers that were unclear about their meaning [whether they were confronting or not] or expressed suspicion about the game or the manipulation; These were excluded from analyses as noted in “Participants and procedure”), and disagreements (*n*’s < 10 across studies) were discussed. For confronting, coders were asked to identify those responses which expressed a verbal disagreement, pointing out the prejudicial nature of the comment, and/or asking the perpetrator to stop making similar comments. Non-confronting was defined as no reply/empty textbox, or replies in a non-confronting manner (e.g., “OK”, “if that’s what you feel”), or endorsement of the remark/ agreement with the biased player. For examples of responses across studies see Table 1.

**Table 1:** Examples of responses (confronting and non-confronting) to the prejudiced player across studies.

	Confronting	Non-confronting
Study 1a	Come on man are you seriously being this racist on a survey?? grow up and read Nah dude, you're a racist and a jackass, considering he was doing better than player A Why do you say that? You said that because he is black, didn't you! That is wrong dude! People like you is what is wrong with this country. Just because someone has a different color of skin doesn't mean they are any less than someone with white skin. In fact, I bet if we looked into your background we would find something about you, wouldn't we! Your an idiot! I'm not racist and I WILL NOT play with someone who is!	I understand I suppose so?
Study 1b	SUCH comment is not necessary. be kind That sort of thinking is disgusting and pure racism I don't believe that. I know some Muslims and they are good people I think you are making a generalized statement and coming across as a bit ignorant or prejudiced. There are many good Muslims, just like there are many good Christians	Lol No comment
Study 2	[translated] You are so rude That’s really racist This was not a nice message Jesus please don’t say things like this How can you judge without knowing a person?	As you wish OK Do as you see best fit
Study 3	good god man! I beg of you, reevaluate your prejudices He's a person just like you and me racist faggot. Latinx are more intelligent than your bitch ass that's messed up and racist That's a nasty thing to say and you should be ashamed of yourself	I hear what you are saying. Everyone is entitled to their opinions Maybe so Lol No comment

#### Hypothetical confronting

As a lead-up we first asked participants in the hypothetical condition at the end of the survey if they witnessed any insult during the game, but regardless of their response in the next page they were told that we are asking this because another survey respondent reported about a possibly prejudiced player, and we described the prejudiced situation exactly as it occurred in the *actual condition* (also the slur was quoted; for script see appendix on OSF)—and we asked if they witnessed something like this. Regardless of their answer in the next page we asked: “If you would have witnessed this incident, do you think you would have confronted the Picker player in the message after the observing session?” on a slider from 0 = I would’ve not confronted him to 100 = I would’ve totally confronted him.

## Results

**Figure 2:**
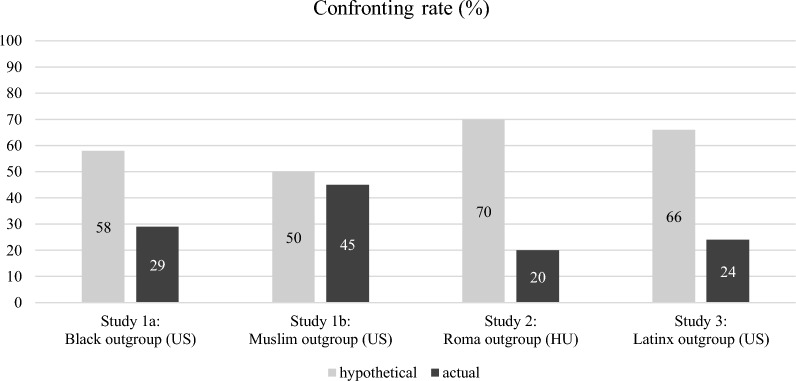
Confronting rates across studies (in percentages).

The coding of the responses to the prejudiced player in the actual condition revealed that 29% (11 out of 38) of participants confronted, 71% have not (empty textbox n = 17, non-confronting replies n = 10). Hypothetical confronting had a mean of 58.14 (*SD* = 36.07, *Mdn* = 65.50). Considering both values as percentage (29% and 58%), probability-comparison analysis (http://vassarstats.net/propdiff_ind.html) indicates a pattern of general overestimation, hypothetical confronting is significantly higher than actual confronting (*z* = -2.64, *p* < 0.01, two-tailed). See Fig. 2 for confronting rates across studies.

**Figure 3:**
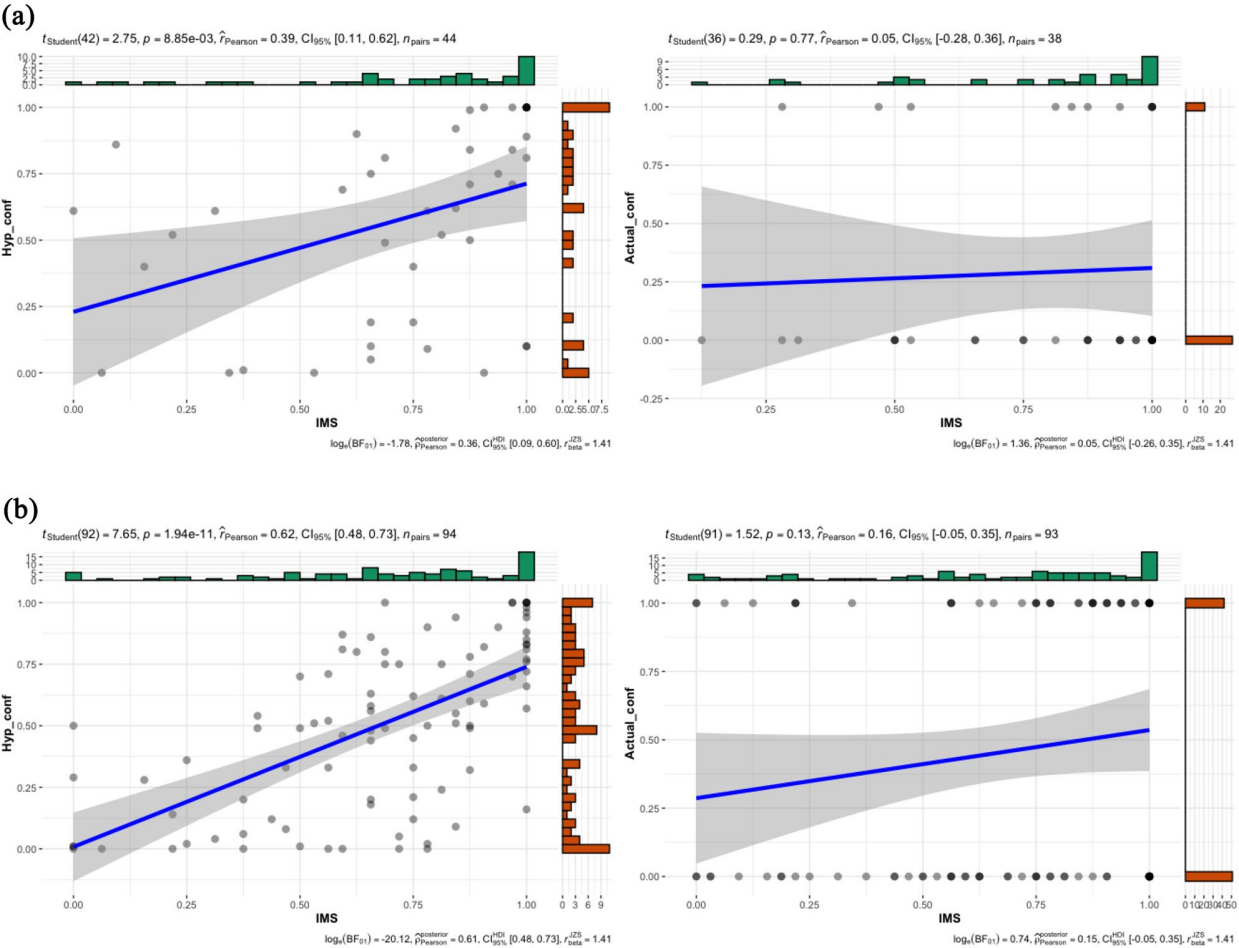

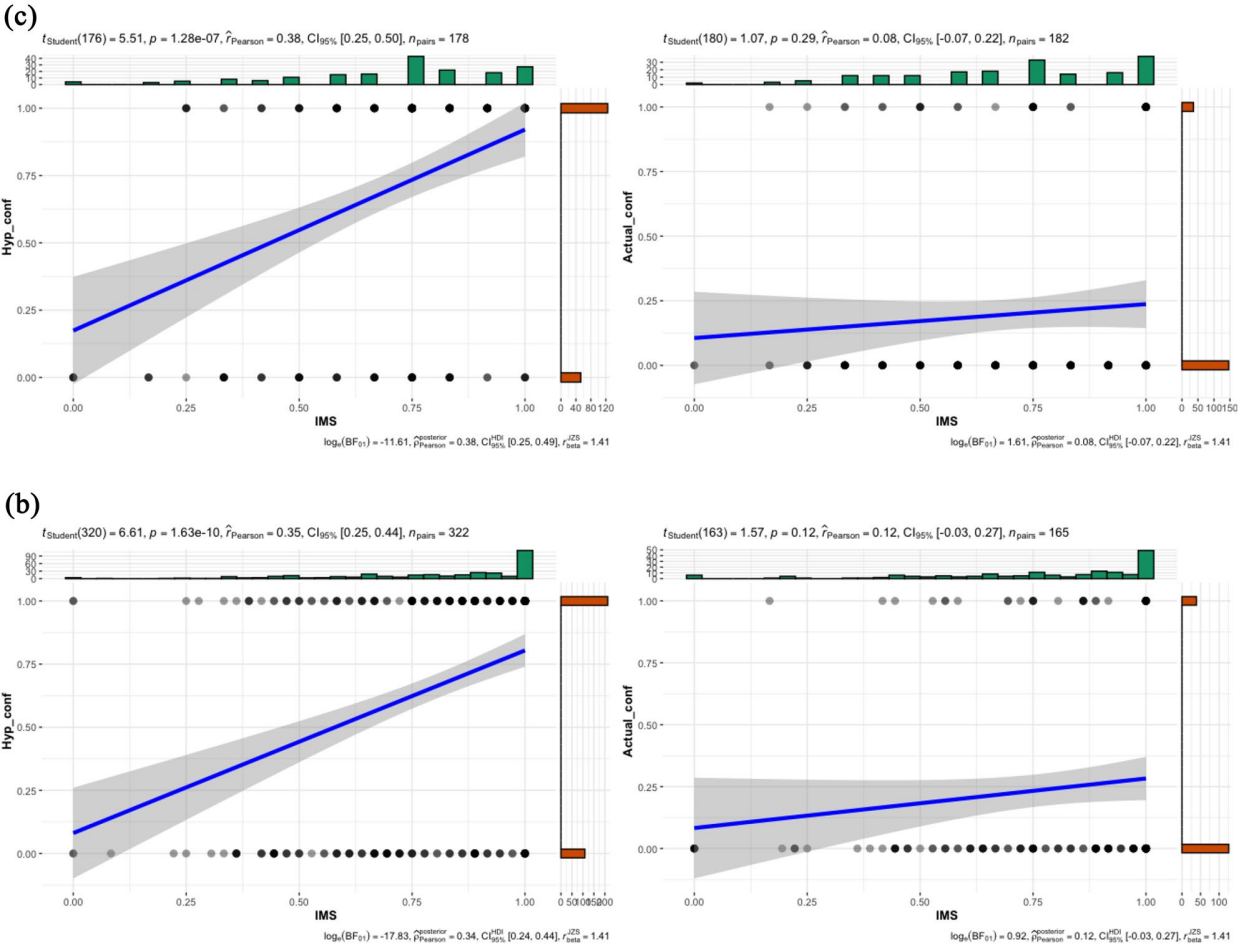
Correlation of IMS with hypothetical confronting and with actual confronting across studies.

To retain consistency across and within studies, all study variables were POMS transformed (proportion of maximum scaling) to a scale from 0 to 1^41,42^. To test our prediction about egalitarian values and confronting, we conducted a linear regression model for hypothetical confronting, and logistic regression for actual confronting with IMS and EMS as predictors. The overall model for hypothetical confronting was significant (*R*^2^ = 0.16, *F*(2,43) = 4.01, *p* = 0.03), and IMS was a unique (positive) significant predictor (*β* = 0.39, *t* = 2.69, *p* = 0.01), whereas EMS was not (*β* = −0.11, *t* = −0.75, *p* = 0.46; zero-order correlation: *r* = −0.13, *p* = 0.41). The overall model for actual confronting was not significant (*χ*^*2*^(2,38) = 2.89, *p* = 0.24, *R*^2^ = 0.07; Cox & Snell R Square reported across studies), and neither IMS (*B* = 0.00, *SE* = 1.48, *W* = 0.00, *p* > 0.99) nor EMS (*B* = −2.15, *SE* = 1.35, *W* = 2.51, *p* = 0.11; zero-order correlation: *r* = −0.27, *p* = 0.10) were significant unique predictors. For visualizing associations with IMS (including values of correlational analysis) see Fig. 3a.

To test whether IMS is significantly more strongly associated with hypothetical than actual confronting, we conducted a Fisher r-to-z transformation test (http://vassarstats.net/rdiff.html) and we did not find a significant difference, *z* = 1.57, *p* = 0.12.

### Discussion and introduction to Study 1b

In Study 1a, we found partial evidence for our hypothesis. We did not find a correlation between EMS and (any) confronting. IMS were associated with hypothetical confronting (those higher on IMS reported higher likelihood of confronting), and it was not associated with actual confronting. However, this difference of association was not significant, possibly because the study was underpowered. In the next studies, we acquired higher sample sizes. Another limitation of this study is that the IMS-EMS scales appeared *after* witnessing prejudice—to avoid revealing the purpose of the game. The following studies were run in two rounds, where IMS and EMS were assessed days or weeks before the witnessing paradigm. In Study 1a, participants were exposed to no bias in the hypothetical condition, in the next study, to allow a closer experience to what occurs in the actual condition, participants were exposed to (some) bias unrelated to racism and they had an opportunity to confront it. The hypothetical confronting question itself still referred to the intergroup prejudice scenario.

## Study 1b

### Method

#### Participants and procedure

We recruited 300 U.S. residents through mTurk (TurkPrime), who completed our first survey (“pre-survey”) for monetary compensation. It included demographic questions (like in Study 1a, including a question about religion) and the IMS-EMS scale (same as in study 1a, but changed to “Muslims” as the outgroup, α = 0.94 for IMS, α = 0.91 for EMS [unlike in study 1a, we only used 3 items for EMS in studies 1b-3]). Five weeks later the same respondents were invited back (except those identified as Arab/Muslim in pre-survey, n = 3) from a different mTurk account, so participants could not know the surveys were connected. With 31% dropout rate, 206 respondents returned (out of which n = 7 did not complete the survey). Participants who failed the attention check in the pre-survey were excluded from analyses (n = 2). We measured suspicion about the credibility of the game or any element of the manipulated scenario with participants’ reply to the biased player, and we excluded those who expressed suspicion about the game (n = 3) or gave unclear answer (n = 7) (see about coding confronting later). Following exclusion, 187 participants remained in the study (50% female, age range: 18–74, *M*_age_ = 36 years, *SD*_age_ = 12.3). They participated in the game, where they were randomly assigned to actual (n = 93) or hypothetical conditions (n = 94).

Participants first completed a survey with the IMS-EMS measures, and few weeks later from another researcher’s mTurk account they received the experimental survey with the witnessing paradigm. This allowed us to minimize the likelihood that participants figure out the purpose of the witnessing paradigm and to ensure that initial egalitarian commitment (self-reporting on IMS-EMS) would not affect their responses in the prejudiced scenario.

For the witnessing paradigm, all participants believed that they are observing a trust-based online game (“Trust game”)—we changed the game to make it simpler and more credible (e.g., we removed profile photos). In the prejudice condition, they witnessed a player being discriminatory and making a prejudiced remark about a Muslim player and they had an opportunity to confront.

Unlike in study 1a, in the hypothetical condition, participants witnessed a player discriminating against another player for his name (not involving racism). The hypothetical confronting question referred to the intergroup prejudice scenario, similarly to study 1a, but it was adapted to the current manipulation, and we asked: “If you would have witnessed this incident, do you think you would have confronted that mentioned player by messaging him?”. As in study 1a, on a slider from 0 = I would’ve not confronted him to 100 = I would’ve totally confronted him. Actual confronting was measured like in study 1a.

#### Witnessing paradigm (trust game)

Participants believed they were observing real players in an online game (and then they would play it themselves). Participants were told the cover story that researchers are testing how being observed and the gender of other players and gender of observers influence players’ trusting behavior, for example if players are more trusting towards women than men. The game was based on the behavioral economic trust game^43^, where Player A decides how much money of an initial endowment to send to Player B. The sent amount is then multiplied by some number and Player B decides how much to send back to Player A. Before starting the game, participants were given instructions and quiz on rules, and we emphasized that the most beneficial behavior is sharing more money with one’s opponent (for instruction see appendix on OSF).

**Figure 4:**
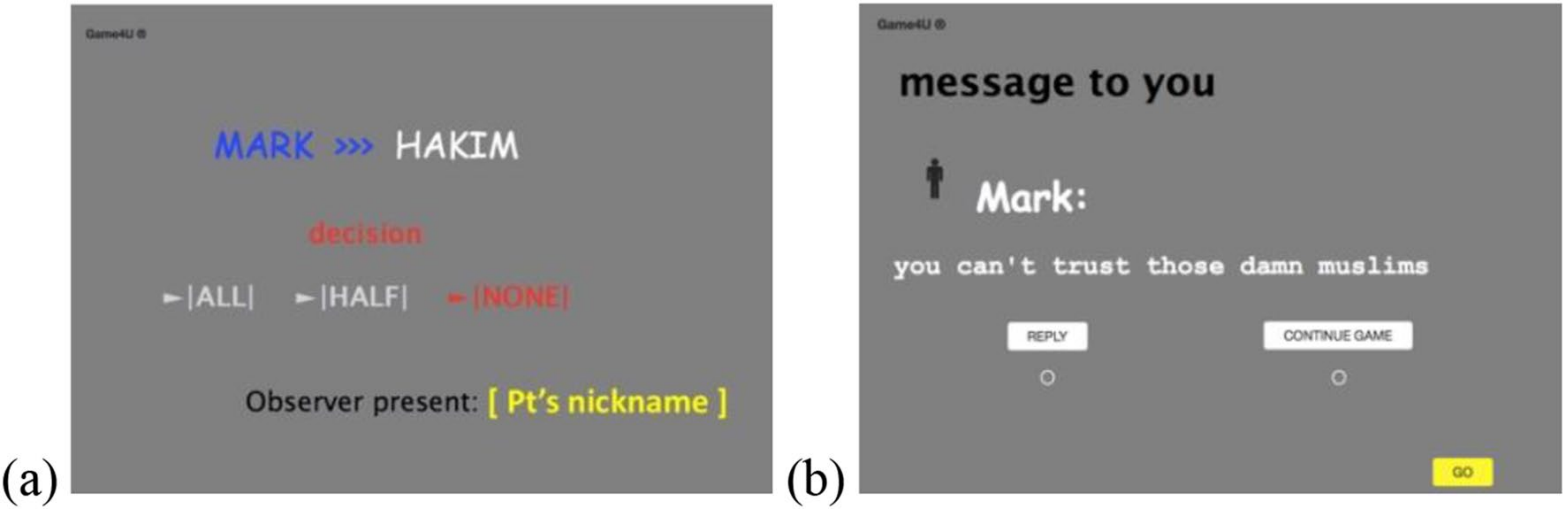
Scenes from the game in Study 1b (Muslim outgroup). Observer is present with their nickname. (**a**) Prejudiced player (Mark) is playing with the Muslim player (Hakim) and offers him no money; (**b**) Mark messages the participant with a prejudiced remark about Muslims.

When participants entered the game site, they were asked to provide their nickname, which appeared throughout the game to make them feel more present in the situation. The names of the other players also appeared on screen. All participants were assigned to observe a player called ‘Mark’ and observed two decoy rounds and exchanged (programmed) messages with Mark. Then, participants in the intergroup prejudice condition were presented with ‘Hakim’ as new opponent to Mark, while participants in the hypothetical condition were presented with ‘Jeff’. In all conditions, participants saw that Mark chose to give no money to Hakim/Jeff (but kept it all to himself)—which was unlike his behavior in the previous observed rounds. Then, Mark privately messaged the participant saying, “You can’t trust those damn Muslims” (actual condition)/“I have a bad feeling about people named Jeff” (hypothetical condition). Under the biased message (across conditions), participants could either press ‘reply’ or ‘continue game’. Those who replied received a notification that the message was read (but received no response from Mark), and the observed game continued. Finally, participants played the (programmed) game themselves for bonus money. For scenes from the game see Fig. 4. Unlike in study 1a, in this game participants knew they would not have future interactions with the prejudiced player, therefore perceived costs of confronting were potentially lower.

### Results

According to the coding of the responses, 45% (42 out of 93) of participants confronted, 55% have not (continued without replying n = 47, non-confronting replies n = 3, agreed n = 1). Hypothetical confronting had a mean of 50.45 (*SD* = 33.11, *Mdn* = 51.50). Considering both values as percentage (45% and 50%), probability-comparison analysis indicated that unlike in study 1a, there was no general overestimation, hypothetical confronting was not significantly higher than actual confronting (*z* = −0.74, *p* = 0.46).

Like in study 1a, all study variables were transformed to a scale from 0 to 1. The overall linear regression model for hypothetical confronting was significant (*R*^2^ = 0.40, *F*(2,93) = 30.19, *p* < 0.001), and IMS was a unique (positive) significant predictor (*β* = 0.60, *t* = 7.21, *p* < 0.001), EMS was not (*β* = −0.10, *t* = −1.25, *p* = 0.22; zero-order correlation: *r* = −0.24, *p* = 0.02). The overall logistic regression model for actual confronting was not significant (χ^2^ (2,93) = 2.68, *p* = 0.26, *R*^2^ = 0.03), and neither IMS (*B* = 0.98, *SE* = 0.70, *W* = 1.98, *p* = 0.16) nor EMS (*B* = −0.47, *SE* = 0.80, *W* = 0.34, *p* = 0.56; zero-order correlation: *r* = 0.08, *p* = 0.44) was a significant unique predictor. The Fisher r-to-z test showed that IMS was significantly more strongly associated with hypothetical than actual confronting, *z* = 3.79, *p* < 0.001 (two-tailed). For association with IMS see Fig. 3b.

### Discussion and introduction to Study 2

In Study 1b, we found that egalitarian values (as indicated by IMS scores) were significantly associated with hypothetical confronting, but not with actual confronting, and this difference was significant. In this study, EMS was negatively associated with hypothetical confronting (although when controlling for IMS, this association becomes non-significant), and not with actual confronting.

In study 1b the confronting rate (45%) was much higher than in study 1a (29%) or compared to findings in previous research^2,4^, resulting in the non-significant overall difference between actual vs. hypothetical confronting (overestimation). This variance in confronting rates could be because of the different outgroups, and the manipulated anti-Muslim slur was more blatant than the anti-Black slur, and participants in this paradigm (unlike in study 1a) had little prospective ramification for confronting. In the next studies, we would use more blatant remarks, but also (re)introduce some cost to confronting. We introduced costs not for the purpose to serve our hypothesis, but to mirror face-to-face interactions, which often involve many costs—especially if the witnessed incident is blatant^44^.

To increase external validity of the findings, Study 2 was conducted in Hungary with Roma minority outgroup (who comprise the largest ethnic minority, around 8–10% of the population)^45^. Roma people face hate crimes and pervasive discrimination in all areas of life^46^. Additionally, we again changed the hypothetical condition to relate even more closely to the actual condition. Participants were exposed to the same exact intergroup prejudice but had no opportunity to confront it. We also changed the scaling of the hypothetical measure (from continuous measure of 0–100 to dichotomous ‘yes’ or ‘no’ like our assessment of actual confronting) to be able to directly compare actual and hypothetical confronting in one analysis (to test an interaction effect).

## Study 2

### Method

#### Participants and procedure

The study was conducted in two rounds at the university, and all study materials were in Hungarian. The pre-survey was completed for course credits by 833 Hungarian students from various disciplines/schools, and it included the IMS (α = 0.90) and EMS (α = 0.72) scales altered to “Roma” as the outgroup. After a few weeks, students were approached from a different researcher and with 48% dropout, 436 participants returned to participate. Following exclusion of those who did not pass the 2 attention check questions in the presurvey (n = 67), those who identified as Roma (n = 1), those who had suspicion (n = 2) or had unclear answer (n = 6) to biased message, 360 participants remained in the study (75% female, age range: 18–53, *M*_age_ = 22.3 years, *SD*_age_ = 4.9). They were randomized in the actual (n = 182) and hypothetical condition (n = 178).

Like study 1b, participants first completed the IMS–EMS measures (pre-survey), a few weeks later they were approached with the game paradigm (“Share game”; post-survey). Participants in the actual condition witnessed prejudice against Roma people and had an opportunity to confront. In the hypothetical condition, unlike in previous studies, participants witnessed the exact same prejudice as in the actual condition but had no opportunity to confront.

We measured hypothetical confronting by asking participants at the end of the survey (among filler questions about the game) whether they experienced bias based on ‘gender’ or ‘else’, and in a next page we asked whether “If you had an opportunity to message him, do you think you would have confronted the player for his behavior toward the minority individual?” with answer options of ‘Yes, I would have confronted him.’ (1) or ‘No, I would have not confronted him.’ (0). This way we could compare the hypothetical confronting to the actual confronting (where we also use dichotomous coding) in the same analysis.

#### Witnessing paradigm (share game)

We slightly altered the game instruction of the Trust game to increase the cost of confronting by telling participants that the observed player will be their opponent, thus if they confront him, he could punish them by not sharing money. Due to this change, we altered the trust game (in which both players give money) to an ultimatum game (there is a giver and a receiver). However, we told participants that they would *not* be a giver to their observed player, they will be a receiver. We done it so participants could not plan to punish (confront) the perpetrator by not returning money to him when they play together, instead of messaging him—which would make measurement of confronting arbitrary. To still make sharing seem like the rational behavior, we explained that “receivers” might become “givers” across rounds, and we called this game the “Share Game”. For instructions see appendix on OSF. To decrease suspicion about certain aspects of the game, (student) participants were made to believe that other participants might be non-students from a survey company or a gaming site.

Another change we made is that the perpetrator messaged participants with a prejudicial slur indicating their intention not to share money with the (minority) opponent, and participants could reply before the perpetrator acted on this plan, so participants’ confrontation was consequential (i.e., they could preventively intervene). The message in both conditions were: “well im not giving money to gypsy thieves” (grammar mistake was intentional).

### Results

The coding of the responses revealed that 20% (36 out of 182) of participants confronted, 80% did not (continued without replying n = 125, non-confronting replies n = 17, agreed n = 4). In the hypothetical condition, 70% of participants indicated confronting intentions (125 out of 178). Probability-comparison analysis indicated a general overestimation, meaning that hypothetical confronting was significantly higher than actual confronting (*z* = −9.62, *p* < 0.001). All study variables were transformed to 0 to 1.

To test our main prediction, we conducted a logistic regression (Hayes’ PROCESS, Model 1, 10,000 bootstrap samples) with condition (hypothetical vs. actual) and IMS (continuous) as predictors of confronting (no vs. yes). As predicted, the interaction between condition (hypothetical vs. actual) and IMS on confronting rate was significant, *B* = −2.84, *SE* = 1.13, *Z* = −2.51, *p* = 0.01, 95% CI [−5.06, −0.62]. The interaction between EMS and condition was not significant, *B* = 0.19, *SE* = 1.09, *Z* = 0.17, *p* = 0.86, 95% CI [−1.95, 2.33]. Correlational analyses, indicating the simple effects, showed that IMS positively and significantly correlated with hypothetical confronting, but not with actual confronting. See Fig. 3c. Additionally, like in Study 1b, Fisher r-to-z test for IMS revealed significant difference, *z* = 3.01, *p* = 0.003, where IMS was more strongly associated with hypothetical than actual confronting. There was no significant relationship between EMS and hypothetical (*r* = −0.09, *p* = 0.23) or with actual confronting (*r* = −0.06, *p* = 0.45).

### Discussion and introduction to Study 3

As predicted, in Study 2 the interaction between IMS and confronting (actual vs. hypothetical) was significant, whereby the more egalitarian participants were, the more they tended to overestimate confronting. Such results were not significant with EMS, suggesting that the overestimation effect is associated with IMS but not EMS. This led us to assume that egalitarians’ pronounced overestimation is unlikely driven by superficial social normative pressure or by hypocrisy in the sense that it occurs among those who are more honestly and internally motivated to control prejudice, and not among those who are motivated for social normative pressure. However, it is still unclear why overestimation is more pronounced among stronger egalitarians, and why they do not fulfill their high confronting intentions.

The goal of Study 3 is to test a potential underlying mechanism for the aversive bystander effect among egalitarians. Based on prior work^36,38^ and considering egalitarians’ potential for optimism bias and threat to the self when witnessing prejudice, we predicted that behavioral uncertainty is associated with less confronting especially for stronger (vs. weaker) egalitarians. We predicted that the actual situation (compared to hypothetical) will be associated with more uncertainty, which in turn will be associated with lower likelihood of confronting.

## Study 3

### Method

We recruited 631 U.S. residents through mTurk (Turkprime). Whites/Caucasians/European-Americans and those who passed a bot check question were able to complete the pre-survey, which included the IMS (α = 0.89) and EMS scale (α = 0.93). One week later, from a different mTurk account, we returned to participants who passed the attention check in the pre-survey. Data was collected for 5 weeks until reaching 485 participants (and additional n = 5 who did not fully complete the survey, but reached the survey question at the end), who would return to participate in the experiment (22% dropout). We excluded those respondents who expressed suspicion in their answer (n = 2) or had unclear answer to the biased player’s message (n = 1), leaving 487 participants in the study (51% female, *M*_*age*_ = 40.8 years, *SD*_*age*_ = 13.3, range: 19–89).

Study procedure was like in study 2 except we added both types of hypothetical conditions from study 1b and study 2, participants were randomly assigned into actual (n = 165), hypothetical-exposure (n = 157), and hypothetical-interpersonal conditions (n = 165).

During the Share game paradigm, in the actual condition, a player (Mark) discriminated against a Latino player (Sanchez) by not sharing money with him and messaging the participant: “yeah like if you could only trust latinos not stealinh our jobs” (typo intentional). Participants in the hypothetical-exposure condition witnessed the same scenario but had no opportunity to reply (i.e., confront). Participants in the hypothetical-interpersonal condition witnessed the same as in study 1b (“I have a bad gut feeling about people named Jeff”).

To measure behavioral uncertainty, we adapted the “respond decision” subscale of the Confronting Prejudiced Responses scale (CPR)^47^. At the end of the post-survey, in the intergroup and hypothetical-exposure conditions, we asked about the observed player’s potential bias similarly to study 2, specifically whether they experienced bias based on gender/religion/ethnicity or else. In the hypothetical-interpersonal condition (like in study 1b) we then reported to them the event that occurred in the actual condition. In the next page, we asked participants whether “Regarding the observed player's [Mark] biased behavior toward a minority individual…” (actual and hypothetical-exposure)/“Regarding the player's biased behavior toward a minority individual that was mentioned above” (hypothetical-interpersonal). This was followed by three statements (α = 0.80) to which participants expressed their agreement (1 = strongly disagree to 7 = strongly agree): (1) I was unsure how to respond to this situation/I am unsure how I would respond to this situation.; (2) I did not know/would not know what to do in this situation; (3) I could think of something appropriate to say [reverse-scored].

At last, in the next page, we measured hypothetical confronting by asking them whether “If you had an opportunity to message him, do you think you would have confronted the player for his behavior toward the minority individual?” (in the hypothetical-exposure condition), or “If you would have witnessed this incident and had an opportunity to message him, do you think you would have confronted the player for his behavior toward the minority individual?” (hypothetical-interpersonal condition), with answer options of ‘Yes, I would have confronted the player for his behavior.’ (1) or ‘No, I would have not confronted the player for his behavior.’ (0).

## Results and discussion

The coding of the responses revealed that 24% (39 out of 165) of participants confronted, 76% did not (continued without replying n = 84, non-confronting replies n = 38, agreed n = 4). In the hypothetical conditions, 68% (107 out of 157) indicated confronting intentions in the exposure, and 64% (106 out of 165) in the interpersonal, averaging both hypotheticals at 66% (213 out of 322). Probability-comparison analysis indicated a general overestimation, hypothetical confronting was significantly higher than actual confronting (exposure: *z* = −8.02, *p* < 0.001, interpersonal: *z* = −7.43, *p* < 0.001, combined: *z* = −8.89, *p* < 0.001). Because the results remain the same if the two hypothetical conditions are handled separately, and there is no significant difference between them, we collapsed their data into one hypothetical condition for ease of reporting and comprehending.

To test our main prediction, the same analyses were conducted as in Study 2. The interaction between condition (hypothetical vs. actual) and IMS on confronting rate was significant, *B* = −2.05, *SE* = 1.00, *Z* = −2.06, *p* = 0.04, 95% CI [−4.00, −0.10]. The interaction was not significant between EMS and condition, *B* = 0.27, *SE* = 0.74, *Z* = 0.37, *p* = 0.71, 95% CI [−1.18, 1.72]. Correlational analysis, indicating the simple effects, showed that IMS was positively and significantly associated with hypothetical confronting but not with actual confronting. See Fig. 3d. Like in studies 1b-2, Fisher r-to-z test for IMS revealed significant difference, *z* = 2.54, *p* = 0.01. EMS negatively and significantly correlated with hypothetical confronting (*r* = −0.13, *p* = 0.02), but not with actual confronting (*r* = −0.09, *p* = 0.25). These results replicated those of Study 2, showing that the more egalitarian participants were, the higher was their tendency to overestimate confronting. We again showed that this overestimation is demonstrated with IMS but not EMS.

**Figure 5:**
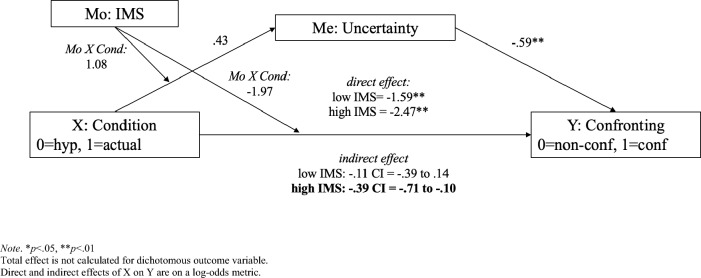
Moderated (IMS) mediation (behavioral uncertainty) in Study 3. *p < 0.05, **p < 0.01. Total effect is not calculated for dichotomous outcome variable. Direct and indirect effects of X on Y are on a log-odds metric.

Finally, we tested a moderated mediation model with bootstrap testing (Hayes’ PROCESS, Model 8), where the IV was condition (hypothetical vs. actual), the DV was confronting, the moderator was IMS, and the mediator was our new variable of behavioral uncertainty (this variable was not transformed). See full report on the OSF page. Simple slopes analyses showed that for high IMS (+ 1 SD), but not low IMS (−1 SD), the bias-corrected bootstrap estimate of the indirect effect had a confidence interval that was reliably different from zero, *B* = −0.39, *SE* = 0.16, 95% CI [−0.71, −0.10]. See Fig. 5. Although the direct effects of condition on confronting remained significant, it is appropriate to interpret the significant indirect effects as mediation (and not as “partial mediation”)^48^. Overall, we found that for stronger egalitarians (but not for weaker egalitarians) behavioral uncertainty mediated the effect of situation on confronting to the extent that in the actual situation (vs. hypothetical) they felt less certainty in how to react which was associated with less bystander confronting.

### Internal meta-analysis

To examine the robustness and reliability of our findings across studies, we conducted internal meta-analysis on the z-test for comparing independent Pearson r’s, which test was consistent across studies. Using CMA software, we meta-analyzed all four studies (N = 1116) and reported them in Table 2. Both random and fixed effects yielded significant effects suggesting that proposed effect (whereby IMS was more strongly associated with hypothetical than with actual confronting) is robust.

**Table 2:** Internal meta-analysis.

Model		Effect size and 95% interval			Test of null (2-tail)		Heterogeneity				Tau squared			
Model	Number studies	Point estimate	Lower limit	Upper limit	Z-value	P-value	Q-value	df (Q)	P-value	I-squared	Tau squared	Standard error	Variance	Tau
Fixed	4	0.993	0.992	0.994	94.061	0.000	347.366	3	0.000	99.136	0.465	0.433	0.188	0.682
Random	4	0.992	0.968	0.998	7.961	0.000								

## General discussion

To test the aversive bystander effect about egalitarian anti-prejudiced values and actual vs. hypothetical confronting, we conducted four studies in two countries among majority members of society with four different outgroups—African Americans in US in study 1a, Muslims in US in study 1b, Roma in Hungary in study 2, and Latinos in US in study 3. We employed an experimental design (study 1b-3) with a behavioral paradigm that allowed us to test actual prejudice confronting behavior. We found support for our predictions across two characteristically different countries (in terms of political system and climate, cultural diversity, minority rights, socio-economic disparities, and geopolitical importance), which strengthens the generalizability of our findings.

In general, we found that in accordance with prior findings^2^, across intergroup contexts, participants’ belief that they would confront prejudice was significantly higher than participants’ tendency to confront when actually placed in the situation (except in Study 1b). Across studies the averaged hypothetical confronting rate was 61% while actual confronting was 24% (excluding the exceptional 45% in study 1b).

According to lay theories about who confronts prejudice—we would think that an individual’s egalitarian values, specifically motivation to avoid prejudice towards an outgroup, should at least partially predict their ally behavior when witnessing prejudice, but our studies show no evidence for that. We predicted that egalitarian values are more reliably associated with hypothetical than actual confronting. Not only we found that they were more strongly related to intentions, but also that egalitarian values did little in determining actual confronting. Across four studies, IMS (i.e., internal motivation to respond without prejudice, which was specified to the outgroup) was not associated with actual confronting. The more egalitarian (self-reported) participants were, the more they overestimated their likelihood of confronting prejudice. Stronger egalitarians (hypothetically) expected themselves more to confront than weaker egalitarians, but in the actual situation, there was little difference in their likelihood of confronting.

These findings align with the aversive racism framework to the extent that non-prejudiced individuals’ ideology and intergroup helping tendency do not necessarily match^19,20^, especially if there is a good enough excuse why to withdraw from helping the outgroup^27^. The situation where someone witnesses prejudice and have an opportunity to confront is a goldmine for aversive racism, for one, because an explicit request for help is not common in these scenarios, and for two, not confronting is an error of omission and not commission. While acting in an inappropriate way (commission) is a red flag for egalitarians, not acting when they should have (omission) is an ambiguous situation that allows for justifying inaction without harming a non-prejudiced self-concept. In the present work, we aimed to go further to test the phenomenon and find possible explanations for what we termed the aversive bystander effect, namely, to investigate how stronger and weaker egalitarians expect themselves to act and how they actually act when witnessing prejudice with an opportunity to confront.

We tested and found that internal motivation to control prejudice (IMS) mattered more in the aversive bystander effect than did the external motivation (EMS). IMS reflects sincere and privately held ideals of oneself as a fair-minded person while EMS stems from perceived public social pressure to adopt nonbiased attitudes and behavior^29^. Our results indicate that egalitarians’ pronounced overestimation is unlikely driven by (superficial) social normative pressure or by hypocrisy in the sense that it occurs among those who are more honestly and internally motivated to control prejudice, and not among those who are only motivated for social normative pressure.

To understand egalitarians’ pronounced overestimation, in Study 3, we tested behavioral uncertainty as an underlying explanation. We found that among stronger egalitarians (but not weaker egalitarians), the sense of inability to identify a response when witnessing prejudice explained the overestimation. Namely, when placed in the actual situation, they felt uncertain what to do compared to the hypothetical situation, and this uncertainty was associated with lower confronting rate. In the aversive racism and justification-suppression frameworks, it is suggested that egalitarians’ prejudiced behavior may be due to hypocrisy, but more so due to unconscious bias, whereby egalitarians are well-intentioned, but their unconscious negative prejudicial feelings and beliefs get expressed in subtle, indirect, and rationalizable ways^18,23,24,31^. However, we chose to focus on behavioral uncertainty because unlike hypocrisy and unconscious bias, it is a malleable buffer to confronting that could be more susceptible to potential intervention.

### Theoretical and practical implications

If egalitarians’ attention is raised to such a buffer to confronting as behavioral uncertainty, given that they are otherwise well-intentioned, and they are provided with tools and methods for confronting—their confrontation could be increased^49–51^. For example, in a study, high-school students were more likely to confront heterosexist bias experienced in school when they previously attended a workshop where they practiced confronting prejudiced remarks, thus were provided with tools for confronting^50^. Similarly, participants in a confrontation role-playing exercise (vs. control group) not only were significantly more likely to confront prejudice, but they also generated more effective confrontation responses^49^.

Our findings provide a grim reflection on intergroup relations research. In empirical studies, confronting prejudice, collective action, and other intergroup behavior should be measured with actual behavior, and not with self-report accounts of intentions. Because not only individuals are bad predictors of their own behavior, but even the relationship of personal values to behavioral intentions was quite dissimilar than values to actual behavior.

Moreover, when designing prejudice reduction or diversity programs in schools, workplaces or generally in the field, one should be aware that promoting egalitarian values towards the outgroup will not necessarily motivate people to act against prejudice in their everyday lives, and while these values predict intentions, it is left for future research to explore how to motivate people to actualize these intentions.

### Limitations and future directions

Our research employed a between-subjects design in terms of measuring hypothetical versus actual confronting. Meaning, similarly to previous studies^2^ we randomly assigned people to the actual or hypothetical condition therefore we do not have individual-level discrepancy value (only the measure of IMS–EMS and the manipulation is tested for each participant). This was done to avoid responses to all measures biasing one another. Additionally, we aimed to measure hypothetical confronting like actual confronting, through the game paradigm, and thus providing participants with both measures would have not been possible. However, individual-level data for the discrepancy would be ideal.

Common methods bias is a valid concern in our research. Namely, both IMS and hypothetical confronting was measured similarly with self-report, as opposed to actual confronting, which was measured behaviorally. Therefore, IMS has a higher likelihood of correlating with hypothetical confronting than with actual confronting. It is unlikely that such bias explains our findings in its entirety, because for one, this did not occur with EMS (it correlated with neither). Secondly, the present research corresponds to the classic finding that attitudes do not (or weakly) predict behavior^52^. While it would have been difficult to measure explicit prejudice with other than a self-report measure, future research should consider using other means to assess initial outgroup attitudes, for example subtle “implicit” measures.

Other individual values than what we measured (IMS–EMS) likely play a role in predicting confronting. In this research, we focused on egalitarian anti-prejudiced values, and specifically on IMS because based on prior studies it could be expected to impact the effects tested in our research. For example, IMS was shown to predict hypothetical confronting^10^, and influence prosocial affective responses to witnessing prejudice^14^, and it is related to the underlying mechanism of aversive racism^16,30^. Additionally, we tailored IMS to the specific outgroup in the given studies rendering it a proxy for a prejudice measure, as well. Regardless of other values that may predict confronting, based on common sense, one would assume that anti-prejudicial values about a minority group should have some impact on the likelihood of confronting prejudice directed towards that minority group. The finding that it is not, is valuable in itself. Nevertheless, future work should focus on individual values or dispositions that may better predict confronting prejudice.

The use of EMS could also be questioned to the extent how it represents any form of egalitarianism at all, even if it is meant to represent external motivation to respond without prejudice^29^. Across studies, EMS was weakly but negatively correlated with both hypothetical confronting (although this was significant only in study 1b and study 3 and became non-significant when controlling for IMS) and had negative but non-significant relationship with actual confronting. This is in line with prior studies showing that EMS is only weakly correlated with having non-prejudiced attitudes, but sometimes even positively (and weakly) correlated with prejudice^35,53^. Interestingly however, in our research, when analyzing those high on EMS (1 SD above the mean) they showed moderately high (hypothetical) intentions of confronting (around 60–65%), therefore we cannot conclude that they were not generally motivated to indicate that they wish to control prejudice.

While conducting research online offers several advantages, such as alleviating ethical concerns, enabling the feasibility of conducting multiple studies across various intergroup contexts, obtaining diverse samples, and achieving larger sample sizes, it is important to acknowledge the limitations regarding the external validity and generalizability of our findings, particularly in relation to face-to-face confrontations that occur offline. We assume that the psychological processes underlying the phenomenon being tested are not specific to online contexts and would similarly manifest in offline situations. Meanwhile we contend that examining online situations has its own merit. Not only people spend a considerable amount of time online, but also more interpersonal interactions are becoming virtual, and socio-political discussions and activism are carried out online (Pew Research Center, 2021). Additionally, although we did not wish to focus on this context alone, but prejudice is often expressed in online game settings^54^. Thus, the used game paradigm has relevance to naturalistic forms of social interactions.

Furthermore, we propose that the online game context serves as an adequate test of our hypothesis, examining whether non-prejudiced individuals overestimate their inclination to confront. In fact, the online context is a conservative test of our hypothesis to the extent that people tend to confront much less in offline situations^2^ thus overestimation would be even more emphasized there. Confrontation may be less frequent offline than online due to certain costs that are not present or weaker in online situations (for example, fear of physical retribution). In this regard, to strengthen generalizability to offline contexts, we attempted to create costs for confronting, such as interpersonal (e.g., losing face, or getting an aggressive reply from the perpetrator) and economic (e.g., the perpetrator will penalize confronters by not sharing money with them in the game). Nonetheless, for stronger ecological validity, it is imperative to replicate the observed effects in other online or offline face-to-face contexts in future research.

## Conclusion

We tend to associate certain type of action with certain ideologies, such as assuming that confronting prejudice towards a minority group is associated with egalitarian values. In the present research, we found a weak relationship between an individual’s egalitarian values and their tendency to confront prejudice in a (allegedly) real situation. These findings encourage self-reflection for individuals with egalitarian values who intend to confront prejudice. Individuals might delude themselves that their egalitarian values would drive them to stand up against prejudice, and thus they would not self-reflect and prepare for situational and other factors that might stop them from actually doing so. Additionally, lay theories about who confronts and who does not may result in misjudgment of some individuals who may identify as non-egalitarian, but when placed in an explicitly unfair situation would be just as likely to stand up for a minority group as those identifying as egalitarian.

## Data Availability

The dataset that supports the current research is openly available on Open Science Framework and can be found here: https://osf.io/d6m47/.
